# Vascularized Iliac Bone Graft for Complex Closure During Spinal Deformity Surgery

**DOI:** 10.1097/GOX.0000000000002345

**Published:** 2019-07-24

**Authors:** Edward M. Reece, Anjali C. Raghuram, Erica L. Bartlett, Tyler T. Lazaro, Robert Y. North, Michael A. Bohl, Alexander E. Ropper

**Affiliations:** From the * Division of Plastic Surgery, Baylor College of Medicine, Houston, Tex.; † Department of Neurosurgery, Baylor College of Medicine, Houston, Tex.; ‡ Department of Neurosurgery, Barrow Neurological Institute, St. Joseph’s Hospital and Medical Center, Phoenix, Ariz.

## Abstract

Spinal pseudarthrosis is failure of bony union across a segment where arthrodesis has been attempted. A variety of treatment approaches have been proposed to promote bony union, including use of vascularized bone graft. We report a successful application of vascularized pedicled iliac crest bone graft (ICBG) to treat recurrent lumbar and sacral pseudarthrosis. The vascularized ICBG offers the advantages of biocompatibility, mechanical stability, and minimal antigenicity. Vascularized bone transfers to the spine undergo fusion without resorption, even in the presence of compressive and rotatory forces. With its ease of access within the operative field and limited donor site morbidity, vascularized ICBG is an ideal orthobiologic that expedites time to bony union.

## Abstract 2

Supplemental Digital Content is available in the text.

Spinal pseudarthrosis, or failed solid bony union, is a well-known complication of spinal fusion procedures.^1^ A variety of treatment algorithms to address symptomatic pseudarthrosis have been described in the literature, but failures are not uncommon. There is a growing body of evidence that the addition of vascularized bone graft can aid, and even expedite, time to bony union through osteogenic and osteoinductive properties of vascularized bone.^2^

The most common source of vascularized bone involves the fibula. However, an alternative source of vascularized donor bone is from the iliac crest. We herein describe the technique and clinical application of vascularized pedicled iliac crest bone graft (ICBG) to facilitate bony union for recurrent spinal pseudarthrosis.

## SURGICAL TECHNIQUE

The iliac crest connects the trunk with the lower extremities. It is supplied by the deep circumflex iliac vessels and lumbar segmental vessels. It is further supplied by the periosteal attachments of the intervening musculature, including the quadratus lumborum and paraspinous muscles.^3^ Vascularized iliac crest can be harvested by preserving all periosteal attachments to an intervening segment, which can then be rotated into a defect (**see figure**, **Supplemental Digital Content 1**, which displays an illustrative representation of the rotation of pedicled vascularized iliac crest for repair of spinal defects, **http://links.lww.com/PRSGO/B148**).

The spinal defect is measured and markings are transposed onto the posterior iliac crest. It is imperative that all periosteal attachments are maintained to ensure adequate vascularity of the graft. A corticocancellous segment of bone is osteotomized with a reciprocating saw. Three bicortical osteotomies are performed, medially, laterally, and inferiorly, with care taken not to plunge after the second cortex is passed, as the retroperitoneum lies immediately beneath [**see figure**, **Supplemental Digital Content 2**, which displays an osteotomized segment of iliac crest attached to the quadratus lumborum muscle: (A) midline; (B) ICBG; (C) longissimus thoracis muscle; (D) the iliac crest; (E) quadratus lumborum muscle, http://links.lww.com/PRSGO/B149].

The bone segment is then grasped, and the gluteus maximus and iliacus are separated from the graft. The bone segment is freed and separated from the paraspinous muscles [**see figure**, **Supplemental Digital Content 3**, which displays an osteotomized segment of iliac crest separated from the paraspinous muscles: (A) midline; (B) ICBG; (C) longissimus thoracis muscle; (D) the iliac crest; (E) quadratus lumborum muscle, http://links.lww.com/PRSGO/B150].

The graft is rotated and translated under the paraspinous muscles into the defect for osteosynthesis [**see figure**, **Supplemental Digital Content 4**, which displays rotation of the ICBG under the longissimus (paraspinous) muscle: (A) midline; (B) ICBG rotated under the longissimus muscle; (C) longissimus thoracis muscle; (D) the iliac crest; (E) quadratus lumborum muscle, http://links.lww.com/PRSGO/B151] [**see figure**, **Supplemental Digital Content 5**, which displays rotation of the ICBG into the midline defect: (A) midline; (B) ICBG rotated under the longissimus muscle; (C) longissimus thoracis muscle; (D) quadratus lumborum muscle, http://links.lww.com/PRSGO/B152] (See Video, [online], which demonstrates the technique for harvesting pedicled vascularized bone graft).

**Video 1.** This video demonstrates the technique for harvesting pedicled vascularized bone graft.

## CASE REPORT

**Fig. 1.**
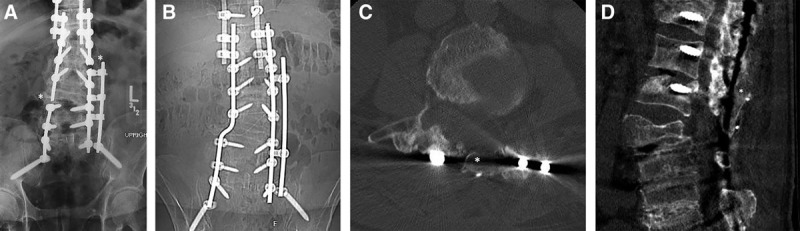
Preoperative and postoperative imaging of a patient undergoing application of vascularized pedicled iliac crest to treat recurrent lumbar and sacral pseudarthrosis. A, Preoperative anteroposterior (AP) lumbar spine x-ray demonstrating bilateral rod fractures (*) with underlying pseudarthroses. B, One year postoperative AP lumbar spine x-ray with revised spinal hardware and faint bone graft over the mid-lumbar spine. C, Postoperative computed tomography (CT) scan, axial cut, at the L3 level showing the cortical edges of the iliac crest bone graft (*). D, Postoperative CT scan, sagittal cut, with the bone graft partially obscured by the hardware artifact (*).

The patient is a 74-year-old, highly functional man with lumbar and sacral pseudarthrosis after 3 previous failed spinal procedures. Imaging demonstrated multilevel compression deformities within the lower thoracic and lumbar vertebrae and bilateral rod fractures with pseudarthrosis at L1–L2 and L3–L4 (Fig. 1A). He underwent revision surgery for a T11 to pelvis fixation and posterolateral arthrodesis. Given his multiple previous spinal procedures and failures, vascularized pedicled iliac crest was elected to enhance and expedite union. This was performed immediately following spinal hardware revision in the prone position.

A 2 × 6 cm bicortical segment of posterior iliac crest was osteotomized, at least 1 cm from the posterior superior iliac crest, with care to maintain the quadratus lumborum attachments. The pedicled bone graft was tunneled under the oblique musculature and into the defect where the facets and remaining lamina were decorticated, spanning L2–L4 and fixed in place under compression with titanium cables (Fig. 1B–D). Bleeding was visualized from the graft edges, which confirmed vascularity. Additional allograft material was placed around the graft. Paraspinous muscle flaps provided durable soft-tissue coverage over the construct, and the skin was closed over 2 drains. The patient was mobilized with a thoracic lumbar sacral orthosis (TLSO) brace postoperatively. No significant complications were encountered. Imaging revealed integration of bone graft into the spine at 1 year (Fig. 1B–D).

## DISCUSSION

Spinal pseudarthrosis should be suspected in any patient presenting with recurrent pain and/or neurologic symptoms in the follow-up period after spinal fusion surgery. In patients with clinically suspected pseudarthrosis, suggestive radiographic findings include implant failure, hardware fractures, radiolucencies, and deformity.^4^ Given the unpredictability of functional results after revision surgery for spinal pseudarthrosis, there is growing interest in preventive measures and treatment approaches for pseudarthrosis to improve spinal fusion rates.

The ideal orthobiologics offer osteogenic, osteoinductive, and osteoconductive potential.^5^ A variety of bone graft materials have been described in the literature, including autologous grafts, bone marrow aspirate allografts, demineralized bone matrix, ceramics, and bone morphogenetic protein-2.^5–7^ Autologous grafts include cancellous and cortical bone grafts; the former has increased porosity and offers faster revascularization and greater biologic activity, whereas the latter provides greater initial strength and stability, but with decreased vascular ingrowth potential.^5,6^

Often considered the gold standard among autologous grafts, the ICBG is recognized for its biocompatibility, mechanical stability, and minimal antigenicity.^5^ An alternative to microsurgical bone transfer, the pedicled ICBG retains the benefits of vascularized bone without the contributing difficulty and morbidity of a free tissue transfer. Notably, in the presence of compression and rotatory loading, vascularized bone transfers to the lumbar spine undergo integration and fusion without resorption. They maintain greater radiologic density than nonvascularized bone grafts and have adequate blood supply.^2^

The vascularized ICBG, based on intervening muscular attachments, offers an approach to fill and revascularize bone defects from the lower thoracic spine to the sacrum and pelvis and from the lower sternum to the thoracic cage.^8,9^ The senior author has used this technique for spinal reconstruction in 12 patients; however, this concept of maintaining perfusion by periosteal attachments has been applied to other areas, such as the hand. Benefits of the ICBG include a donor site within the operative field and ease of elevation. After the osteotomy, cancellous bone graft is readily available from within the pelvis for additional grafting. Last, use of vascularized autografts allows active infections to be treated in situ, obviating the need for larger revision procedures to prevent sequestra.^10^ There is limited donor site morbidity other than potential for a small palpable step-off in thin patients. Additionally, seroma is not uncommon given the extensive field of dissection, and thus drains are mandatory.

Given the curvature of the posterior crest, a maximum length of 6 cm of corticocancellous bone can be harvested. Increased patient age, osteoporosis, and active smoking increase the complication profile of this procedure. The addition of vascularized ICBG increases the operative time, albeit by <1 hour. This technique shows promise as an adjunct for facilitating fusion and should therefore be recognized in the armamentarium for spinal reconstruction.

## SUMMARY

Vascularized bone grafts have been shown to expedite time to bony union, especially in cases of spinal pseudarthrosis. Vascularized ICBG offer an adjunct to spinal reconstruction procedures by maximizing potential for solid bony union and providing a reasonable alternative to free bone transfer. This technique follows consistent anatomy, ease of harvest within the operative field, and minimal donor site morbidity. For patients with complex spinal pathology or previous fusion failure, the vascularized ICBG offers a rescue or salvage strategy and should be an available tool in the reconstructive surgeon’s armamentarium for spinal fusion.

## Supplementary Material
