# Supplementary figures and images for: Vascularized Iliac Bone Graft for Complex Closure During Spinal Deformity Surgery
Source: Plast Reconstr Surg Glob Open. 2019 Jul 24;7(7):e2345. doi: 10.1097/GOX.0000000000002345 (PMC6952161; doi:10.1097/GOX.0000000000002345)

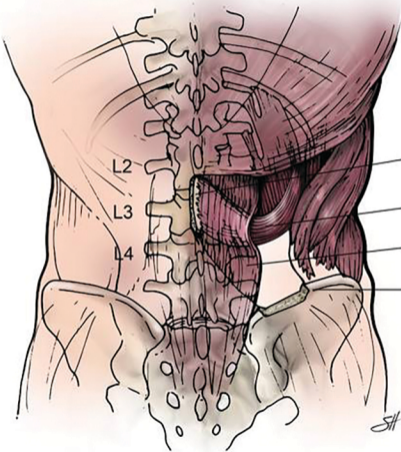

Bone graft

Quadratus lumborum m.

Longissimus thoracis m.

Iliac crest

SH

Supplement: Supplementary file 2 [file gox-7-e2345-s002.pdf]

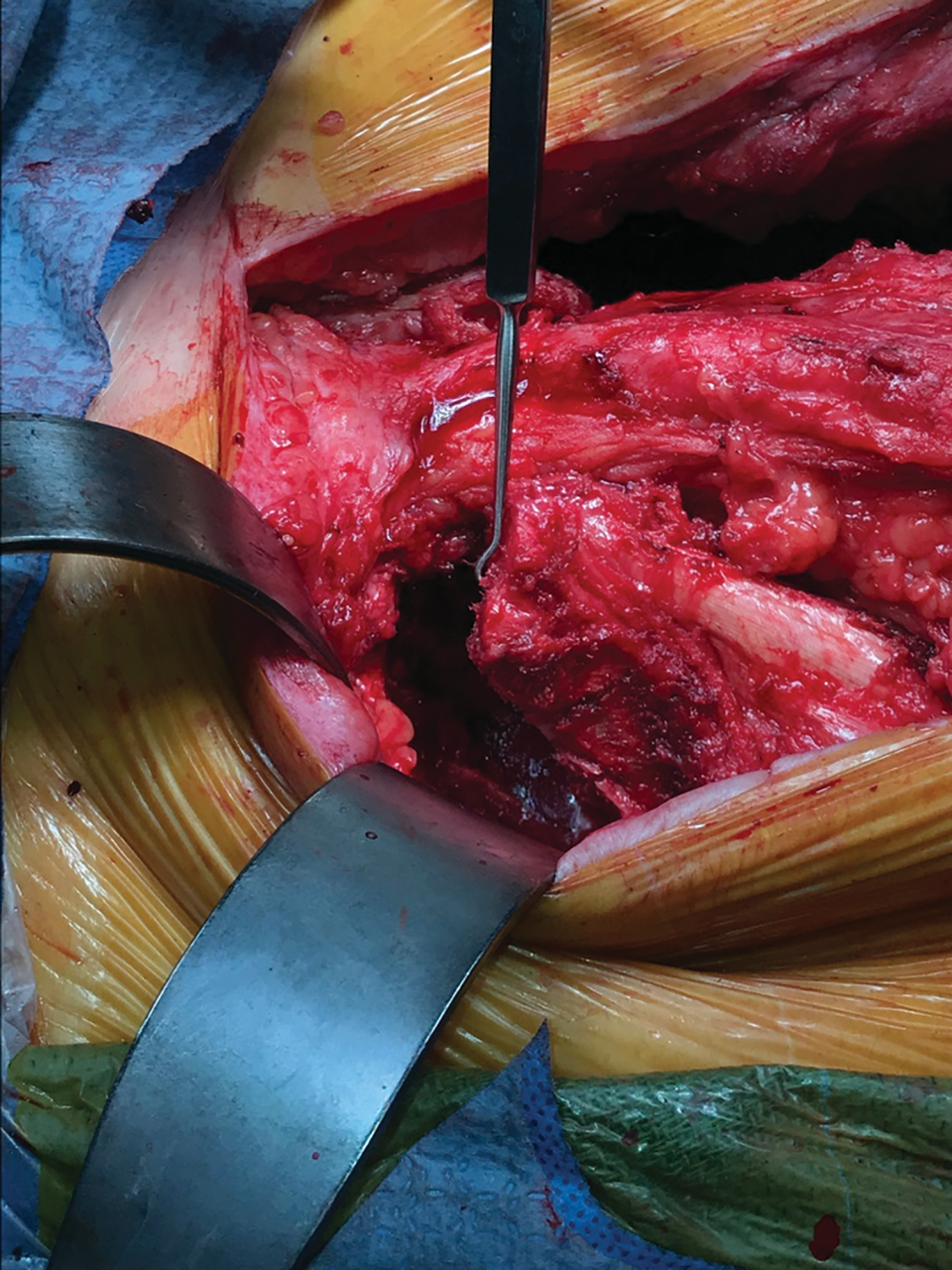

Supplement: Supplementary file 3 [file gox-7-e2345-s003.pdf]

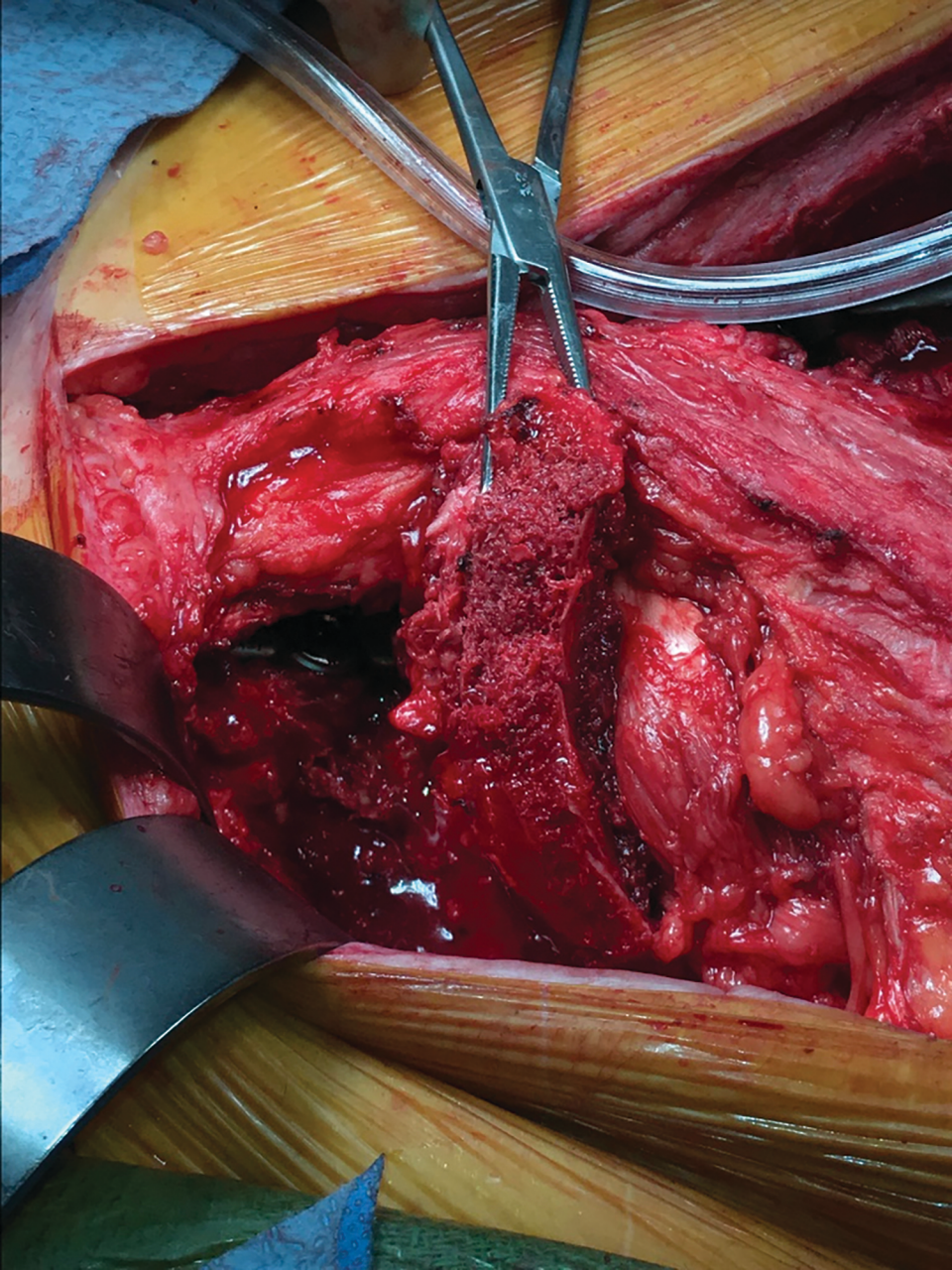

Supplement: Supplementary file 4 [file gox-7-e2345-s004.pdf]
